# The diminishing polyads of parenting: exploring cultural perspectives of ‘good parenting' using grounded theory analysis

**DOI:** 10.3389/fpsyg.2026.1894854

**Published:** 2026-09-07

**Authors:** Giulia Ciuffo, Marta Landoni, Martina Smorti, Stefano Bianchi, Chiara Ionio, Sergio A. Silverio

**Affiliations:** 1 Dipartimento di Psicologia, Università Cattolica del Sacro Cuore, Milan, Italy; 2 Dipartimento di Patologia Chirurgica, Medica, Molecolare e dell'Area Critica, Università di Pisa, Pisa, Italy; 3 Dipartimento di Scienze Biomediche e Cliniche, Università degli Studi di Milano, Milan, Italy; 4 Department of Psychology, University of Liverpool, Liverpool, United Kingdom; 5 Department of Women & Children's Health, King's College London, London, United Kingdom

**Keywords:** fatherhood, grounded theory, motherhood, parental beliefs, parenting, qualitative research

## Abstract

**Introduction:**

This study investigates Italian parents' perceptions of ‘good parenting' and cultural variations. 250 mothers and 87 fathers were surveyed during the third trimester, with follow-ups at 6–12 weeks and 6 months postpartum.

**Methods:**

Using Grounded Theory Analysis, data underwent iterative coding, forming lower and higher order codes, super-categories, and themes.

**Results:**

The resulting theory, ‘The diminishing polyads of parenting,' identifies four themes for mothers and three for fathers. Mothers prioritize emotional presence, self-sacrifice, and role balance, while fathers focus more on physical presence and active involvement.

**Discussion:**

The study advocates for a gender-neutral approach to supporting diverse family dynamics, recognizing evolving parental views and societal norms' impact. Inclusive interventions and policies can better aid families in their parenting journey.

## Introduction

In recent decades, parenthood has been the subject of a variety of studies. To understand how parents structure their parental identity and the parenting strategies they adopt, it is important to examine how parents conceptualize and construct their perception of parenthood. In different countries, people may adhere to a unique cultural framework characterized by different values, ideologies, norms, and beliefs (Yang, 2018). These cultural contexts help to establish norms and define competencies which are esteemed (Markus and Hamedani, 2020), and serve as a benchmark for appropriate actions, attitudes, and contextual relevance in shaping the construction of the self and identity formation (Harkness and Super, 2021; Super and Harkness, 2020a).

This even applies to parenting culture. All parents have ideas about the optimal behaviors and qualities necessary to be and act as “*good parent*s” (Weaver et al., 2020). A growing body of literature suggests ‘*ideal-parenting beliefs*' are a global phenomenon and parents in different cultures rely on such beliefs to fulfill their parenting responsibilities (Pedersen, 2016; Super and Harkness, 2020b). Whilst certain elements of these beliefs are considered universal, such as the expectation of parental care; the exact definition of what it means to be a ‘good parent' appears to vary by cultural contexts (Bornstein, 2012, 2013; Choate and Engstrom, 2014; Lo Cricchio et al., 2019). What is considered ideal in one cultural milieu may be perceived as inappropriate in another (Choate and Engstrom, 2014; Li, 2012). For example, in a recent study comparing data from 37 countries (Lin et al., 2023), distinct sets of ideal-parent beliefs were identified across five different parenting culture zones. Asian parents prioritized being responsible and family-focused, whilst African parents emphasized responsibility and proper demeanor. Hispanic-Italian parents valued being loving and responsible. In the final two zones—Western I and Russian (i.e., Poland, Russia, Australia, Serbia) and Western II (i.e., France, Belgium, Switzerland)—the predominant themes remained consistent as the most significant elements, yet there were some subtle variations: English-speaking, EU, and Russian parents tend to prioritize being caring, while French-speaking ones emphasized the importance of listening or being present. Moreover, in the Italian context, it has been suggested that a ‘*good parent*' seems to be the one who spends time with their children and fosters their social development (Bombi et al., 2015), and raises them in a safe and nurturing family environment (Bembich, 2016). Nevertheless, it has been found that when describing their beliefs and practices related to good parenting, Sicilian mothers tended to prioritize control, discipline, and firmness as essential elements, placing them above warmth and responsiveness (Lo Cricchio et al., 2019).

The definition of ‘*good parents*,' therefore, must also take into account historical, political, and economic factors which have prevailed in Western countries over time. Indeed, prior to the advent of neoliberal, political, economic, and cultural changes, family dynamics in Western industrialized nations were subject to clearly defined norms, in which mothers were primarily entrusted with unpaid care and household tasks. As a result, women with children were increasingly encouraged or forced to pursue paid work. Whilst this change has led to a blurring of the traditional societal roles of mothers and fathers (Knight and Brinton, 2017; Sullivan, 2021), the division of labor and responsibilities within families has remained largely gendered (Grunow and Evertsson, 2019; Schmidt et al., 2019; Valiquette-Tessier et al., 2019). In this context, life history theory (King, 2015) offers valuable insights into the dynamics of parental decision-making and resource allocation within families. Just as life history theory describes the allocation of resources across an organism's lifespan in response to environmental cues, parental decisions are influenced by historical, political, and economic contexts. For instance, the shift toward mothers entering the workforce reflects an adaptation to changing socio-economic environments, where the allocation of resources—both time and effort—is recalibrated to meet evolving demands. Moreover, the persistence of the gendered division of labor underscores the enduring influence of societal norms and expectations, shaping parental roles and responsibilities. Indeed, despite these changes, mothers are still generally rated as “*good*” because they invest time and effort in caring for their children (Ennis, 2014). These beliefs serve as a compass for parenting behavior (Hale et al., 2017), and influence parental decision-making and the fulfillment of parental responsibilities in both routine and demanding situations (Lo Cricchio et al., 2019; Weaver et al., 2020).

Furthermore, the relevance of these cultural, political, economic, and societal variables is such that parents may experience feelings of guilt, self-stigma, self-criticism, and occasionally despair when they perceive themselves as falling short of their parenting ideals (Eaton et al., 2016). For example, extensive research has highlighted the damaging effects of the ‘*motherhood myth*' ideology, which places mothers under immense pressure and leads to stress, isolation, and lasting negative effect on mental health (Liss et al., 2013; Staneva and Wigginton, 2018). This ideology places the sole responsibility for the development, health, and wellbeing of children on women, and ignores the potential contribution of fathers.

Studies on maternal guilt have shown it often stems from unrealistic expectations of perfect motherhood (Rotkirch and Janhunen, 2010), with mothers being disproportionately blamed for various aspects of their children's lives, including their actions, behaviors, and health (Jackson and Mannix, 2004). In response to these societal pressures, some mothers adopt practices of ‘*Intensive mothering*,' and strive to fulfill ideals of motherhood, often leading to feelings of guilt when these expectations are unattainable (Arendell, 2000; Rizzo et al., 2013). Other, seminal work challenges the notion of motherhood as a purely joyful and fulfilling experience, revealing the social, emotional, and economic impacts that accompany the transition to motherhood (Nicolson, 1999). Indeed, as stated before, societal expectations of motherhood often overlook the realities faced by women, leading to feelings of isolation, resentment, and powerlessness.

As these beliefs are crucial in shaping parenting cultures, researchers have recently recognized the exploration of these beliefs as a promising avenue in educational science (Super and Harkness, 2020b). In psychological research, a common method of describing cultural differences in parenting is to adopt established theoretical frameworks. Whilst this allows for comparative analysis between communities or countries, the direct application of such frameworks in specific cultural contexts may unintentionally overlook relevant indigenous concepts or processes (Lansford et al., 2016; Yang, 2018). One possible solution to this challenge is to apply a bottom-up exploratory approach. By directly engaging with the understanding of ‘*good parenting*' which people have in each culture, and without pre-supposing a particular framework, this approach has the propensity to reveal culturally specific core concepts which might otherwise be neglected through the imposition of pre-conceived notions.

For these reasons, we employed a grounded theory analysis to investigate how Italian parents conceptualize ‘*good parents*' and how this conceptualization may differ from other cultures and from previous studies in Italy.

## Methods

### Study design

This study was part of a wider international project, INTERSECT (International Survey of Childbirth-related Trauma), conducted by the Trauma Psychology Research Unit of the Catholic University of the Sacred Heart in Milan and the Center for Maternal and Child Health Research of the City University of London. The project had a longitudinal design with three waves of data collection.

### Study team

The authors comprise a multidisciplinary team of researchers and clinical academics specializing in Psychology (GC, ML, CI, MS, SAS), Clinical Sciences (SB), and Social and Implementation Science (SAS), focusing on maternity and perinatal mental health. Data oversight was provided by a psychologist with expertise in qualitative research (SAS), while analysis was spearheaded by two psychologists (GC and ML) with extensive experience in both clinical practice and academia. Regular meetings were conducted among analysts to deliberate on emerging themes and evolving theories, with each stage of analysis vetted by the wider team for validation. Employing bracketing (Gearing, 2004) enabled researchers to adhere to the Grounded Theory principle of ‘no a priori assumptions' (Glaser and Strauss, 1967; Silverio et al., 2019), recognizing the challenge researchers face in maintaining neutrality when closely engaged with their subject matter or study population (Thornberg and Dunne, 2019).

### Theoretical perspective

This study aligns with gendered lifecourse research (Silverio, 2021), particularly focusing on the normative life course of women in Western contexts, such as Italy, which includes the significant transitions of pregnancy and childbirth. These transitions into parenthood offer valuable opportunities for empirical investigation. Grounded Theory Analysis, as advocated by Glaser and Strauss, recognizes the importance of transitions in life trajectories, each with distinct trajectories and roles shaping one's life path (Glaser and Strauss, 1967). Our philosophical framework is rooted in critical realist ontological and objectivist epistemological domains (Levers, 2013), with a critical approach to reflexivity and an acknowledgment of the subjectivity and objectivity within the research team, some of whom have children and some do not. This approach establishes a post-positivist research paradigm (Levers, 2013), where participants' narratives are regarded as authentic representations of their lived realities.

### Ethical approval

Ethical approvals were jointly sought and granted from the Catholic University of the Sacred Heart [ref: 05-22], the University of Pisa [ref: 05-22], and the S. Giuseppe Hospital in Milan [ref: 550/2022] Research Ethics Committees. Prior to commencing the survey, participants were asked to read an information sheet and electronically sign a consent form.

### Recruitment and participants

We recruited 250 mothers (*M*_age_ = 33.7), in the third trimester of pregnancy; and 87 fathers (*M*_age_ = 35.6) recruited at the time their partner was in the third trimester of pregnancy. Fathers could participate if their partner chose not to participate.

In the first phase (T1), participants were recruited via birthing centers, hospitals, or clinics and assisted to complete the questionnaire via Qualtrics. They were then sent a follow-up e-mail at 6–12 weeks postpartum (T2), and finally six months after the birth (T3). To minimize self-selection bias in the sample, participants were not recruited via social media or other on-line methods. Inclusion criteria were: (i) participants were in the third trimester of pregnancy; (ii) at least 18 years of age; (iii) gave informed consent to participate, and (iv) had a good knowledge of the Italian language.

Most participants lived in Lombardy (60.2%), or Tuscany (31.8%), and the remainder was distributed throughout Italy (8.0%). For those who provided marital status data, most participants were cohabiting (49.9%), were married (46.3%), with very few who were single (0.6%) or divorced (0.6%). Mothers either had a post-graduate (14.2%) or an undergraduate education (40.1%), a secondary (34.4%) or middle (6.2%) school diploma, or other qualifications (3.9%), as their highest level of qualifications, with only very few having no qualifications (1.3%). A minority of mothers were unemployed (5.7%) and most were primiparous (66.3%).

Fathers had a a post-graduate (11.7%) or an undergraduate education (34.0%), a secondary (38.8%) or middle (10.7%) school diploma, or other qualifications (3.9%), as their highest level of qualifications, with a minority having no qualifications (1.0%). No fathers were unemployed.

Due to attrition from the study, T2 data collection saw a reduction in participants (*n* = 83 mothers; 16 fathers); and likewise, T3 analysis is based on fewer participants still (*n* = 41 mothers; 8 fathers). We therefore view the later phases of the project data—particularly in the fathers' data – as more exploratory than explanatory.

### Data analysis

The analysis focused on the participants' answers to the open question “*What is a good mother/father for you?*.” Classical grounded theory analysis (Glaser and Strauss, 1967) was utilized allowing for inductive and iterative coding, analysis, and interpretation. Whilst not a usual choice for survey data, where there is richness in data and novelty in investigation (such as in this research), the exploratory nature of grounded theory can be helpful, hence its selection here. Grounded theory analysis relies on a series of well-established principles: having no *a priori* assumptions of the data or population; conducting ‘data-driven' or ‘data-heavy' analysis; coding data *in vivo* using the words of participants as initial codes; all whilst ensuring reflexive practice ran through all aspects of data collection, analysis, and interpretation. We also engaged with the principle of constant comparison between transcripts throughout the analysis.

To begin with ‘line-by-line coding' was undertaken, followed by ‘focused coding' [GC; ML]. The focused codes were then merged into ‘super-categories' (or emergent ‘lower order themes'). Final themes were then identified with the assistance of a more experienced researcher [SAS], who also guided how to take account of the temporality of longitudinal data (Silverio et al., 2019). During the analysis process, we interrogated codes, super-categories, themes, and theory through a comprehensive review known as an ‘intra-team defense' which involved in-depth questioning and discussion of theory between team members from different disciplines (Silverio et al., 2019). Theoretical saturation was accepted when analysis data from additional survey responses failed to unveil any novel concepts (Holton and Walsh, 2016; Vasileiou et al., 2018). Given the iterative process of the analysis, the issue of data saturation was first addressed within the team whereby it was found that variation was generally due to different naming practices rather than semantic differences in what was being described (Guest et al., 2006). Thematic diagrams were instrumental in generating theory and directing the ultimate formation of the theory. In refining the grounded theory, the research team deliberated on the theory to craft a cohesive narrative outlining the observed processes and patterns depicted in the thematic diagrams.

## Results

**Figure 1:**
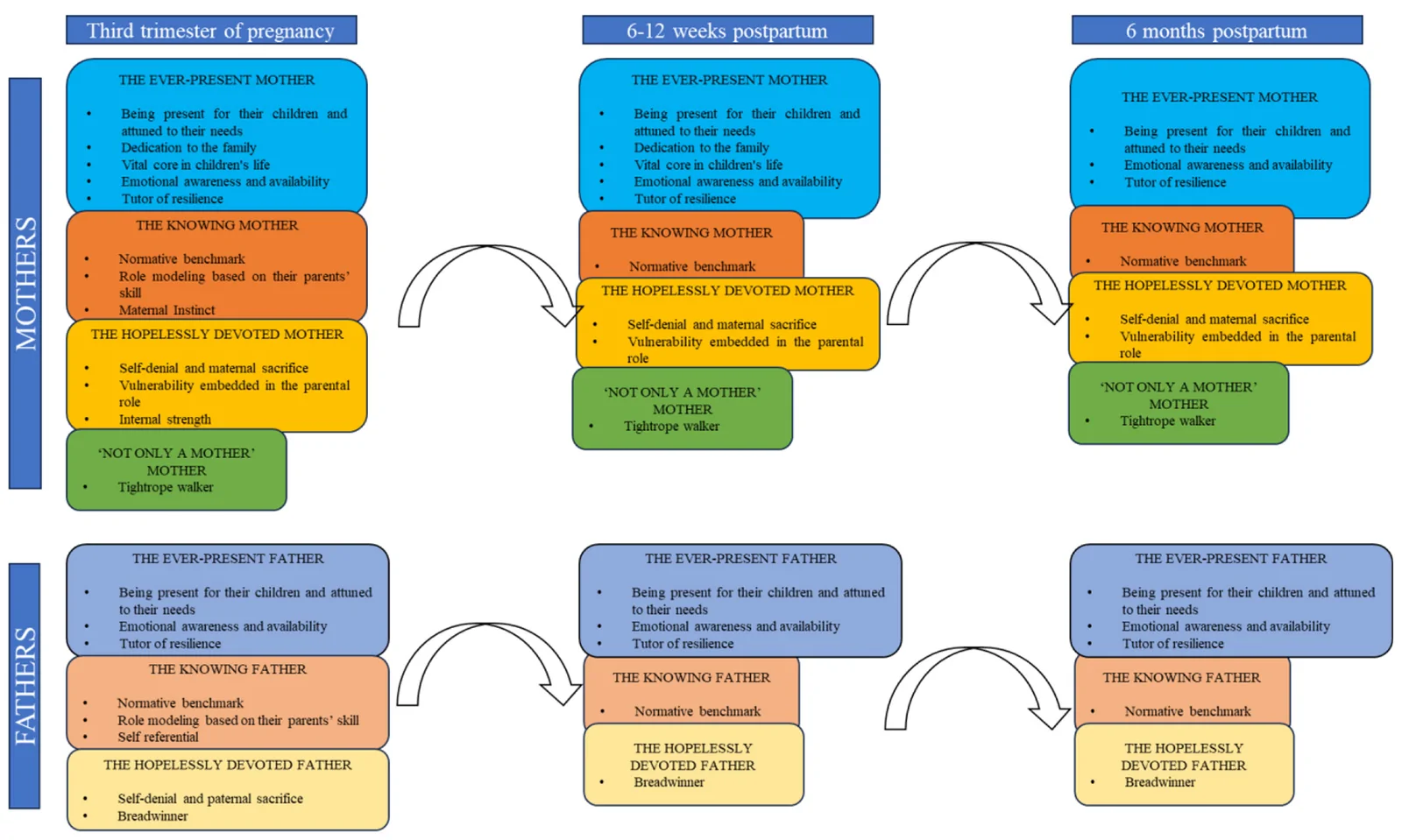
Thematic diagram of super-categories.

Analysis led to the emergence of four themes for mothers and three themes for fathers. Each theme is presented below for both mothers and fathers, with the most illustrative quotations. Figure 1 summarize themes and related super-categories in each assessment wave for both mothers and fathers. The following paragraphs illustrate the themes with the most representative quotations. Further quotations can be found in Table 1.

**Table 1:** Representative quotations for each theme.

Mothers		Fathers	
Theme	Quotations	Theme	Quotations
The ever-present mother	“*A mother who is present in good times and bad, who fulfills her child's wishes”; “A mother who is present, who can listen and support”; “A mother who takes care of her children”; “So many things... she cares for the child, educates the child, understands the child, listens to what her child says and understands when something is wrong even without speaking”; “The one who takes care for her child”; “A person who is able to understand the child's needs and respond appropriately”; “Who can take care of the needs of her family”; “The one who takes care of the family, the house”; “A good mother is the one who takes care of her family”; “Essential for life”; “Tender, loving, caring”; “A mother is loving, caring, affectionate person”; “She must be able to give affection to her child”; “conveys emotions and positive experiences for living together”; “...and teaches him/her to be what is good for him/her”; “A good example of humanity”; “and teaches principles and values to cope better with life”; “… and gives him/her the tools to build the life he/she wants”*.	The ever-present father	“*... must be present”; “A person who knows how to accompany [his child] in his growth”; “The one who gives attention and care to the child's growth”; “... and by no means try to lack anything essential”; “...who conveys unconditional love and affection”; “...the son must feel respected, supported, loved and valued by the father himself”; “...a person who loves”; “Show love in gestures and words”; "...who is able to show love”*.
The knowing mother	“*...and raise him/her to civilly respectful”; “...but also be strict when necessary”; “...and not justify any behavior or action of her son/daughter”; “the one who educates”; “firm in her decisions”; “My mum“; “to be like my mum”; “I want to be like my mum was to me”; “A good mother is the one who does not need to wonder”; “There is no such thing as good or bad mothers”*.	The knowing father	“*The one who educates children”; “...respecting the rules of the family and society”; “strict when needed”, “giving simple and clear rules and principles for living together with others”; “… and corrects the child”; “less flexible than the mother”; “mine”; “my dad”; “Being like me”*.
The hopelessly devoted mother	“*A good mother is someone who puts the welfare of her children first”; “A mother who puts herself second place”; “A person who is willing to do anything for her child”; “The mother who would give her life for her child”; “capable enough”; “......although this is not always easy”; “who discusses and often wonders if he is doing a good job”; “„, despite her fallibility”; “A person who is aware of making mistakes but tries her best”; “...with her limitations”*.	The hopelessly devoted father	“*The one who does everything for his child”; “A person who takes care of the family and does his best to fulfill the basic needs and requirements”; “the family carer”; “who provides for his family in every possible way”*.
The “not only a mother” mother	“*A person who takes care of herself and her child”. “A woman who feels good about herself first and foremost”*		

### Mothers' theme 1: the ever-present mother

For these women, the good mother is the one who is primarily present in her child's life, who is attentive to her child's needs and can pick up on and interpret her child's signals to respond to all her child's needs. This mother is the one who takes care of the child. She is so receptive that she does not even need words. In addition to the needs of her child, she must be ready and attentive to the needs of all family members and respond accordingly. Indeed, she is devoted and committed to her family and home.

“*… and also manages to take care of the house”*

“*A mother who pays attention to the welfare of her family.”*

The good mother is the one who is vital and indispensable in the life of her child, which is why she is and must always be present. She is the one who “has always been there and will always be there.”

“*She is my everything! She is the one who gives you life!”*

“*She has always been there, and will always will be there.”*

She is the most important source of love and care for the child, she is very aware of her own feelings and those of her child. She is able to express love verbally, show affection through actions, and understand the child's emotional needs. She should be nurturing. She should be attentive, and emotionally connected to her children and create a positive and supportive environment for their growth and wellbeing.

“*Loving and affectionate, able to say even a simple ‘I love you' to her child and ask him/her if he/she is happy or if something is wrong”*

“*I think that loving one's child, in the sense of caring, nurturing, living in his emotions and according to his natural inclinations, belongs to the emotional sphere.”*

In addition, a good mother is also able to guide her child toward personal fulfillment and moral development. She is a positive example of humanity, conveys solid values and principles and gives the child practical tools for. She helps the child to overcome challenges, while celebrating successes, and providing comfort in the face of setbacks.

“*...and teaches him/her to be a good person with solid values and principles”*

“*helps him/her to overcome difficulties, celebrates his/her achievements and gives comfort in a setback.”*

### Mothers' theme 2: the knowing mother

For these women, the good mother is the one who is able to set boundaries, who is firm when necessary, and who does not tolerate inappropriate behavior. She guides her children responsibly so that they become responsible individuals in society. She is firm and unwavering figure in upbringing of her child, committed to instilling values which promote respectful and responsible behavior.

“*...and teach her children the right from the wrong and make them responsible members of society.”*

Their knowledge may come from the experiences of their own parents, who can be a role model for shaping their own parenting skills:

“*my role model is definitely my dad...so I should probably be like he was and is to me with my baby”*

“*be aware of what she went through so she does not put her child through similar experiences.”*

Or it could also be intuitive and instinctive. There is no ‘good' or ‘bad' mother, every mother acts on her own love. Inner balance, and personal values. The good mother does not have to question herself about it.

“*I don't think there is a standardized stereotype of a ‘good mother' I think every mother moves through her own love, her own inner balance and according to the values she believes in.”*

### Mothers' theme 3: the hopelessly devoted mother

For these women, a good mother is one who prioritize the needs of her children and puts them above her own needs and desires. These mothers emphasized qualities such as sacrificing personal wellbeing, career aspirations, and even life itself for the good of the child. The core element is unwavering devotion and a willingness to do whatever it takes to ensure the happiness and safety of the child. Essentially, the good mother is described as someone who places her children's needs above all else, demonstrating unconditional love and devotion in her actions and decisions.

“*A mother who puts the child above her needs (money, sleep, health, career)”*

“*A mother who puts her children above all else, who does not sacrifice them for herself.”*

The good mother struggles with feelings of vulnerability and self-doubt, but endeavors to be capable and do her best despite her imperfections. She often questions her abilities and worries about making mistakes, acknowledging her limitations. Despite these challenges, she remains true to her role as a mother and overcomes difficulties by demonstrating resilience and constantly striving to improve herself.

“*A vulnerable woman who constantly feels inadequate and is constantly afraid of making mistakes”*

“*A good mother is the one who always tries to do her best even though sometimes it may not be enough.”*

Indeed, the good mother overcomes any obstacle or challenge to ensure the happiness of her child. She does everything she can to work for her child's wellbeing and happiness, even metaphorically ‘climbing mountains.' A good mother is characterized by her boundless love and her willingness to make sacrifices to support and nurture her child, no matter what difficulty or adversities arise along the way.

“*...but who would overcome any obstacle for her child”*

“*is the one who climbs mountains to make you happy.”*

### Mothers' theme 4: the ‘not only a mother' mother

Although the smallest theme, an important one arose for maternal identity. For these women, the good mother is the one who is able to find a balance between her social role as mother, as a woman and as partner.

“*...not forgetting the importance of not neglecting yourself and your partner”*

“*A mother who is able to take care of herself and her relationship.”*

They emphasize that a mother must prioritize her own wellbeing and maintain a healthy balance between caring for herself, her child, and her partner.

“*She must be able to find a balance between being a mother and being a partner.”*

This includes maintaining a positive self-image, taking care of her personal needs, and actively investing in her relationship with her partner.

“*She is willing to have a sincere and genuine relationship and not an ideal one where I have to sacrifice myself at all costs.”*

“*A mother is also prepared to maintain a relationship with her partner without neglecting him.”*

### Fathers' theme 1: the ever-present father

For these men, the good father is the one who is always present in his child's life. He is always available and has an open ear for the child's needs and growth. The good father is portrayed as someone who is constantly present and supportive. This presence is portrayed as essential to fostering a strong bond and the child's development and wellbeing.

“*An ever-present figure for the child.”*

He gives his children unconditional love and affection. He gives the child genuine care, respect, and support; he makes a point of expressing love and affection and makes the child feel valued, respected and appreciated.

“*...above all, a person who can convey the right affection”*

“*...a father who loves his son, the rest should be a consequence.”*

Based on this, the good father prepares his children for adulthood and supports and encourages them on their way. He recognizes the child's individuality, strengths and limitations. He encourages open communication and the expression of feelings. A good father is the one who guides and supports his child through life's challenges, offering both practical guidance and emotional reassurance. He serves as an example of a peaceful and fulfilling life while maintaining a strong and supportive bond with his child.

“*Be present in good and bad times in everything the child asks for and support him in everything”*

“*A good father is the person who always stands by his child, in good times and bad.”*

### Fathers' theme 2: the knowing father

For these men, the good father plays a significant role in educating and disciplining their children. They emphasize the importance of instilling respect for familial and societal rules, sometimes requiring strictness when necessary. The good father is portrayed as providing clear guidelines and principles for living harmoniously with others, whilst also correcting the child's behavior when needed. Compared to the mother, he may be perceived as less flexible in his approach to discipline and rule enforcement. His knowledge may come from the experiences of one's parents, who can be a role model for the structuring of her own parenting skills. Or the good father could serve himself as a role model for their children and may embody the qualities he hopes his children will emulate:

“*my own father”*

“*what I'd like to become.”*

### Fathers' theme 3: the hopelessly devoted father

For these men, a good father is someone who is selflessly devoted to his children and shows an unwavering commitment and willingness to protect and provide for them at all costs.

“*The one who loves his son so much that he gives his life for him.”*

The good father is a protector and provider for his family. He prioritizes the needs of his partner and children and shows a sense of responsibility and commitment to nurturing the family dynamic, as well as a willingness to sacrifice and take responsibility to support his loved ones.

“*...it is natural for him to take care of his family in a practical way”*

“*A person who thinks of the family and children and puts everything in place.”*

## Discussion

**Figure 2:**
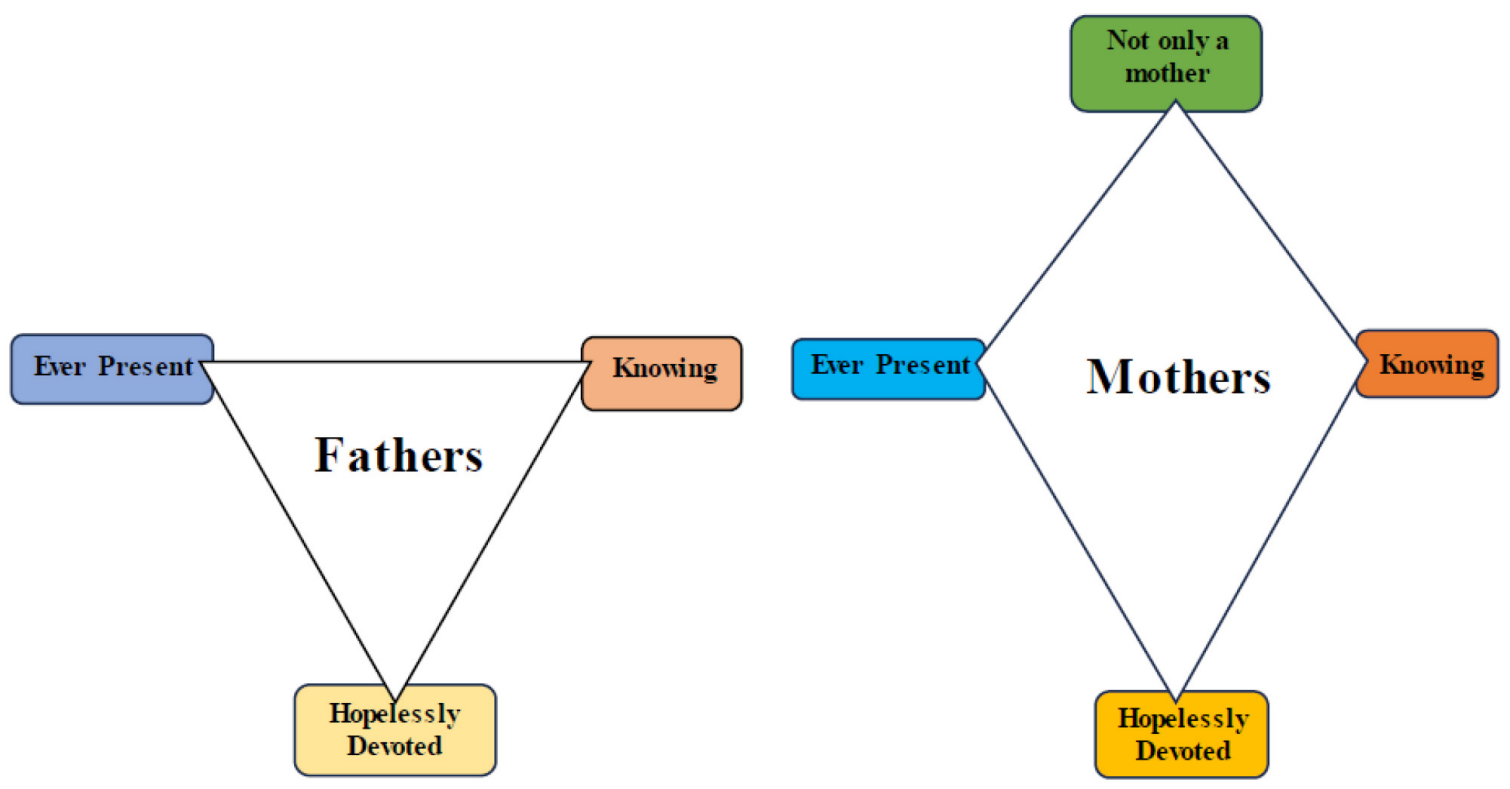
Diagram of the emerging themes.

Taking the emergent themes together, a theory emerges of changing views of good parenthood. Where Italian mothers report a broader, more holistic view of ‘good motherhood,' they also discuss the attempt to reclaim identity outside of motherhood, which potentially is lost during pregnancy and the early postpartum. Fathers, however, do not have this additional facet to their reporting of ‘good fatherhood,' perhaps because their role outside of fatherhood is more clearly defined and not threatened by the physical transition which their female counterparts experience when becoming a parent and the fact that the early survival of their baby does not rely on their physical body. In both mothers and fathers, the reporting of ‘good parenthood' narrows to a core of aspects—possibly explained by the fact that as their baby grows, both mothers and fathers realize some of the idealized aspects of being a ‘good parent' are in fact unnecessary. Again, this is more prevalent in mothers than it is fathers, and we propose this may be because mothers tend to be the primary caregivers, and so their perceptions of parenthood and what is needed from them as parents is more dynamic and adaptable than their male counterparts who may have a less plastic view. Overall, we call this theory: ‘The diminishing polyads of parenting' (Figure 2). This is explained in two ways: Firstly, the idea of having different polyads representing the aspects of ‘good parenthood' for mothers (represented by the kite of interactions for four themes) and fathers (represented by the triangular interaction of three themes) suggests there is a difference in the perception of what parenthood is for each parent; and secondly, the diminishing aspect informs us that the facets of parenting becoming smaller as time with their newborn draws on, potentially for the reasons suggested above. The concentration of these themes over time was evident even in spite of the attrition of parents over the three waves of the study, leading to us suggesting the focus of parents narrows over time as they adopt, embrace, negotiate, and/or embody their new parental role.

Looking at the themes that have emerged from our work, it is clear that Italian mothers have a more holistic and complex view of ‘good parenting' compared to fathers, which tends to remain relatively stable in the early stages of parenthood. The prevailing norm refers to the ideal of the good mother, who is always present, emotionally available, and attentive to her child's signals and needs (The ever-present mother). This norm is reflected in the research findings on mothers' efforts to observe, understand and fulfill their children's signals and preferences (e.g., Afflerback et al., 2013; Byrt and Dempsey, 2020). Mothers feel obliged to be physically present and available and make personal sacrifices to fulfill this societal norm (the hopelessly devoted mother; Carter and Anthony, 2015). As suggested by Schmidt et al. (2022), this notion is reinforced by concepts such as ‘*mother knows best*' and the portrayal of the mother as the bearer of innate talents (Cowdery and Knudson-Martin, 2005). These concepts confer authoritarian status on mothers and embody the idealized notion that only the ‘*knowing mother*' (Davis et al., 2019) can provide optimal care over an extended period of time due to her supposedly unique and natural bond with the child (Christopher, 2012; Dillaway and Paré, 2008; Guendouzi, 2006). These norms ultimately place the responsibility for the development, health, and welfare of their children on women (Seagram and Daniluk, 2002). Expectations of mothers have become increasingly demanding. Not only are they expected to prioritize the needs of the ‘*sacred child*' over their personal needs (Johnston and Swanson, 2006), but they are also required to engage in a level of nurturing and developmental activity that is fully consuming. Empirical evidence supports the widespread acceptance of the ideology of intensive mothering (i.e. Constantinou et al., 2021), which is also supported by time-use studies showing that working mothers today spend more time with their children than mothers who stayed at home in the 1960s (Bianchi, 2000). As mothers began to participate in the labor market, a new need emerged for women to combine paid work with caring for children and the home (The ‘not only a mother' mother).

Rather than viewing paid employment as an integral part of motherhood, mothers are expected to reconcile their professional endeavors with their maternal responsibilities by skillfully balancing these two areas of life (Schmidt et al., 2022). Numerous studies have expressed the expectation that a working mother will not be hindered in her family responsibilities or reduce the time and effort she devotes to her children by her work commitments (Leigh et al., 2012; Liss et al., 2013; Pedersen, 2012; Wainwright et al., 2011). As previously mentioned, there is evidence that mothers often experience predominantly adverse emotional reactions when confronted with conflicting normative expectations, which can jeopardize their overall wellbeing (Baker, 2019; Carroll and Yeadon-Lee, 2021; Constantinou et al., 2021; Forbes et al., 2020). Indeed, mothers from different social backgrounds report ongoing emotional distress resulting from these pressures, which appear to extend throughout the journey of motherhood, continuing into the later stages of life (Gunderson and Barrett, 2017; Mansvelt et al., 2017).

On the other hand, despite some similarities, fathers have a narrower idea of ‘good parenting' than mothers, and this tends to be even narrower in the early stages of parenthood. As with mothers, the theme which emerges most frequently and remains relatively stable over time is the father's physical presence, his emotional availability, and his active role in the child's life (the ever-present father). These findings are in line with previous research (Lin et al., 2023) which characterized Italian parents as ‘loving and responsible.' Fatherhood in Western cultures has undergone a significant ideological change in recent decades. The concept of fatherhood has broadened to encompass practices traditionally associated with ‘mother work' (Burnett et al., 2013; Ewald et al., 2020). As women have increasingly entered the workforce, societal expectations have emerged that men should actively participate in childcare, leading to an increase in fathers' involvement with their children, regardless of whether mothers are employed or not (Pleck and Masciadrelli, 2004; Pedersen, 2012). Modern fatherhood has left behind the traditional breadwinner model which prevailed in the 1950s, in which gainful employment and childcare were equated (Townsend, 2010). Instead, a more complex discourse has developed that emphasizes committed care and ‘involved fathering' (Petts and Knoester, 2018; Ewald et al., 2020). While modern fathers are still primarily expected to support for their families financially (the hopelessly devoted father), they are also increasingly expected (Hochschild and Machung, 2012; Shirani et al., 2012) and desired (Halle et al., 2008; Williams, 2008) to actively participate in the daily tasks and responsibilities of childcare. Consequently, the modern father is perceived as someone who is both financially responsible for his children and actively participates in routine caregiving duties. However, as fathers lack the representation of the last theme, it is clear their identity as a person is not as threatened as mothers are. Breakwell's theory suggests that individuals construct their identities through various social roles and relationships, including those of parenthood. According to Breakwell (1986), individuals are motivated to maintain a positive self-concept, and threats to their identities can lead to psychological discomfort or defensive reactions. The absence of representation of the theme suggests that fathers may not perceive these aspects as central to their parental identity. Consequently, their identity as individuals may not be as closely intertwined with their role as caregivers compared to mothers. Finally, the theme of the knowing father seems to be the legacy of an idea of fatherhood that has characterized Italian fathers for several decades, who, unlike the more ‘*flexible*' mothers, were entrusted with the role of educating their children and imposing rules on them. This is in line with the findings of Lo Cricchio et al. (2019), who found that control, discipline and a sense of entitlement were prevalent in a sample of Sicilian mothers, taking precedence over warmth and responsiveness. As the authors suggested, these themes may be particularly pronounced in the context of southern Italy, which has retained its rural and agricultural character despite the rise of capitalist society.

While we address and acknowledge the differences, it is also important to recognize the similarities in the construction of ‘good parenting' by fathers and mothers. This is in line with previous research (Lin et al., 2023), whose findings point to the existence of ‘*parenting*' cultures worldwide, rather than delineated ‘mothering' and ‘fathering' cultures. Indeed, some authors (Fagan et al., 2014) suggest that the field should adopt a gender-neutral approach to conceptualizing the dimensions of parenting, as there is insufficient evidence to support the uniqueness of the constructs of fathering and mothering. The authors' argument is supported by three sets of findings. First, several studies suggest that the constructs of fathering and mothering are indistinguishable (i.e., Lin et al., 2023). Second, there is evidence that fathers' parenting behaviors have a similar impact on child outcomes as mothers' parenting behaviors (Cabrera et al., 2011). Finally, fathers and mothers are increasingly similar in their roles, the behaviors they exhibit toward children, and the time they invest in childcare (i.e., Europe; Raley et al., 2012). A gender-neutral approach to parenting has several implications for understanding and supporting diverse family structures, including those led by Trans and/or Non-Binary (TNB) parents (Bower-Brown, 2022). First, it encourages researchers to examine parenting behaviors and dynamics without rigid gender stereotypes, allowing for a more nuanced understanding of parenting roles within these families. By acknowledging that gender identity does not dictate caregiving abilities or roles, researchers can explore how TNB parents navigate parenthood in unique ways, free from the constraints of traditional gender norms. Furthermore, a gender-neutral approach emphasizes the importance of addressing fathers' involvement in parenting in both research and clinical practice, recognizing that their influence on child outcomes is comparable to that of mothers. This perspective challenges the notion that caregiving is primarily the responsibility of mothers, promoting a more inclusive understanding of parental roles. By acknowledging and supporting fathers' active participation in parenting, regardless of their gender identity, researchers and practitioners can better support the diverse needs of families. Finally, adopting a gender-neutral approach to parenting underscores the need for policies and practices that promote an equal sharing of caring responsibilities between fathers and mothers, regardless of their gender identity. This shift toward shared parenting responsibilities contributes to greater gender equity within families, challenging traditional gender norms and fostering more equitable relationships between parents. By promoting policies that support all parents in sharing caregiving duties, regardless of gender, society can create more inclusive and supportive environments for all families, including those led by TNB parents.

## Strengths, limitations, and future directions

This study presents several strengths that lay a solid foundation for future research directions. Firstly, it represents a pioneering effort in the Italian context by focusing on both mothers and fathers in exploring perspectives on good parenting. This inclusive approach provides a comprehensive understanding of parenting dynamics within Italian families, shedding light on the roles and beliefs of both parents, which is essential for developing targeted interventions and policies. Secondly, the use of a grounded theory approach is commendable, as it allows themes to emerge organically from participants' responses, free from preconceived notions or theoretical biases. This methodological rigor enhances the credibility and validity of the findings, providing a nuanced insight into how Italian parents conceptualize good parenting within their cultural context. Moreover, the longitudinal design of the study offers valuable insights into the evolution of parental perspectives on good parenting over time, particularly during the critical transition to parenthood. By capturing data across three waves of data collection, the study illuminates how parental beliefs and practices may shift or stabilize in response to the challenges and joys of parenthood. Future research could further explore these longitudinal trajectories to identify key factors influencing parental adaptation and growth. Despite these strengths, the study also faces limitations that point toward fruitful avenues for future research. The regional concentration of participants, primarily from Lombardy and Tuscany, raises questions about the generalizability of the findings to other Italian regions with distinct cultural, social, and economic contexts. Future studies could address this limitation by expanding the sample to include participants from a more diverse range of regions, allowing for cross-regional comparisons to uncover regional variations in parental perspectives on good parenting. Additionally, the reduction in sample size in the second and third waves, particularly among fathers, due to participant dropout highlights the importance of addressing attrition in longitudinal studies. Future research could explore strategies to enhance participant retention, such as implementing incentives, maintaining regular communication with participants, and minimizing survey burden. Further analytic efforts could also be made to track more explicit changes in experience over time, and present data per timepoint, longitudinally with a statistical analytic technique (i.e. Linguistic Inquiry and Word Count) or percentage coverage (i.e. with a content analysis approach) to provide a different way of assessing such data.

In summary, future research in this area could build upon the strengths of this study while addressing its limitations to provide a more comprehensive understanding of parental perspectives on good parenting in Italy, noting our sample was mainly recruited from the Lombardy and Tuscany regions. By employing diverse methodological approaches, expanding the sample to encompass greater regional diversity, and addressing issues of attrition, future studies can contribute to the development of evidence-based interventions and policies to support Italian families in their journey toward effective and fulfilling parenting practices. Furthermore, direct comparisons of partners responses to ‘good' parenting would be an interesting line of inquiry, not undertaken in the present study.

## Conclusion

The study sheds light on how Italian parents conceptualize ‘good parenting' and reveals some gender differences in parental views as well as a refinement of these views over time spent as a new parent, leading to the theory: ‘The diminishing polyads of parenting.' Whilst mothers have a more holistic and complex idea of being a good parent and are confronted with the need to reconcile the different roles they believe they play, fathers have a narrower view that tends to narrow further with the actual transition to parenthood. However, it is crucial to recognize both the differences and similarities in parental perspectives. Research suggests parenting cultures exist worldwide and are not strictly defined by gender. Proponents of a gender-neutral approach argue that the distinction between fatherhood and motherhood is not as clear-cut as once thought. A gender-neutral approach to parenting not only facilitates a deeper understanding of parenting dynamics but also emphasizes the importance of including fathers in both research and practice. This could contribute to the psychological wellbeing of mothers by reducing the pressure they feel to conform to the socially imposed norms and roles of the ‘myth of motherhood.'

## Data Availability

The raw data supporting the conclusions of this article will be made available by the authors, without undue reservation.
